# Differences in Weight Loss by Race and Ethnicity in the PRIDE Trial: a Qualitative Analysis of Participant Perspectives

**DOI:** 10.1007/s11606-022-07521-5

**Published:** 2022-04-25

**Authors:** Rintu Saju, Yelba Castellon-Lopez, Norman Turk, Tannaz Moin, Carol M. Mangione, Keith C. Norris, Amanda Vu, Richard Maranon, Jeffery Fu, Felicia Cheng, O. Kenrik Duru

**Affiliations:** 1 David Geffen School of Medicine, University of California, Los Angeles, CA USA; 2 Department of Family Medicine, University of California, Los Angeles, CA USA; 3 Department of Medicine, Division of General Internal Medicine and Health Services Research, University of California, Los Angeles, CA USA; 4 VA Greater Los Angeles Health System and HSR&D Center for Study of Healthcare Innovation, Implementation & Policy, University of California, Los Angeles, CA USA; 5 Division of Endocrinology, Diabetes & Metabolism, David Geffen School of Medicine at UCLA, Los Angeles, CA USA; 6 Jonathan and Karin Fielding School of Public Health, University of California, Los Angeles, CA USA

**Keywords:** prediabetes, weight loss, shared decision-making, racial and ethnic disparities

## Abstract

**Background:**

Many Diabetes Prevention Program (DPP) translation efforts have been less effective for underresourced populations. In the cluster-randomized Prediabetes Informed Decision and Education (PRIDE) trial, which evaluated a shared decision-making (SDM) intervention for diabetes prevention, Hispanic and non-Hispanic Black participants lost less weight than non-Hispanic White participants at 12-month follow-up.

**Objective:**

To explore perspectives about weight loss from PRIDE participants of different racial and ethnic groups.

**Participants:**

Sample of participants with prediabetes who were randomized to the PRIDE intervention arm (*n*=24).

**Approach:**

We conducted semi-structured interviews within three groups stratified by DPP participation and % weight loss at 12 months: (*DPP+/WL+*, enrolled in DPP and lost >5% weight; *DPP+/WL−*, enrolled in DPP and lost <3% weight; *DPP−/WL−*, did not enroll in DPP and lost <3% weight). Each group was further subdivided on race and ethnicity (non-Hispanic Black (NHB), non-Hispanic White (NHW), Hispanic). Interviews were conducted on Zoom and transcripts were coded and analyzed with Dedoose.

**Key Results:**

Compared to NHW participants, Hispanic and NHB participants more often endorsed weight loss barriers of limited time to make lifestyle changes due to long work and commute hours, inconvenient DPP class locations and offerings, and limited disposable income for extra weight loss activities. Conversely, facilitators of weight loss regardless of race and ethnicity included retirement or having flexible work schedules; being able to identify convenient DPP classes; having a strong, positive support system; and purchasing supplementary resources to support lifestyle change (e.g., gym memberships, one-on-one activity classes).

**Conclusions:**

We found that NHB and Hispanic SDM participants report certain barriers to weight loss more commonly than NHW participants, particularly barriers related to limited disposable income and/or time constraints. Our findings suggest that increased lifestyle change support and flexible program delivery options may be needed to ensure equity in DPP reach, participant engagement, and outcomes.

**Supplementary Information:**

The online version contains supplementary material available at 10.1007/s11606-022-07521-5.

Approximately 160 million US adults are overweight/obese and 88 million have prediabetes.^1, 2^ Since prediabetes and overweight/obesity increase risk of incident type 2 diabetes, both are targets for community and public health interventions.^3–5^ Risk is magnified for patients from under-resourced populations including non-Hispanic Black (NHB) and Hispanic individuals, who are more likely than non-Hispanic White (NHW) persons to be overweight/obese and develop type 2 diabetes.^2, 6–8^

In clinical trials, lifestyle change can reduce diabetes incidence broadly, regardless of race or ethnicity. The Diabetes Prevention Program (DPP) randomized controlled trial, in which 36% of participants were NHB or Hispanic, showed that lifestyle modifications with a 7% weight loss goal led to a 58% reduction in diabetes incidence over 3 years.^5^ Long-term DPP follow-up at 10 and 15 years showed decreased incidence of diabetes by 34% and 27%, respectively.^9, 10^ The American Diabetes Association (ADA),^11^ Centers for Disease Control and Prevention (CDC),^12, 13^ and United States Preventive Services Task Force (USPSTF)^14^ all recommend lifestyle change interventions for overweight/obese individuals at increased risk for type 2 diabetes. Equitable lifestyle change interventions have the potential to attenuate racial and ethnic disparities in diabetes and related complications.

Unfortunately, “real-world” DPP translational studies have generated unequal weight loss by race and ethnicity. In particular, NHB and Hispanic individuals in the DPP lose less weight than NHW participants.^15–18^ Ely et al. document similar findings in the first 4 years of the National DPP, a standardized approach instituted by the CDC providing community-level DPP implementations.^19^ Although participants (*n*=14,747) lost an average of 4.2% body weight, NHW participants lost more weight (4.6%) than those from other racial and ethnic groups, with NHB participants losing the least (3.2%).^20^

We reported results from the Prediabetes Informed Decision and Education (PRIDE) cluster-randomized trial, which used shared decision-making (SDM) to encourage uptake of evidence-based diabetes prevention, including the DPP.^21^ Overall, PRIDE intervention participants were more likely than matched controls to lose weight at 12 months. However, even after adjusting for variables including self-reported income and DPP attendance, NHW patients lost 3.2% body weight on average compared to 1.3% for Hispanic patients and 0.8% for NHB patients.^21^ These weight loss differences by race and ethnicity are consistent with findings from other DPP translational studies.^15–19^

The purpose of the present study was to better understand perspectives on weight loss barriers and facilitators by race and ethnicity in participants who expressed interest in DPP participation after an SDM intervention. Although the primary intent of the study was hypothesis-generating, our goals were to qualitatively explore factors such as adverse social determinants of health that might explain racial and ethnic weight loss disparities and to identify mutable factors for future diabetes prevention efforts in order to promote health equity.

## METHODS

### Description of the PRIDE RCT

PRIDE was a clinic-level cluster-randomized trial conducted from 2015 to 2018 within the UCLA Health primary care network.^22^ Overweight/obese patients with prediabetes (body mass index [BMI] > 24 kg/m^2^ or > 22 kg/m^2^ if Asian; HbA1c 5.7–6.4% within the prior 3 months) from 20 clinics were included. Intervention participants (*n*=515) opted in for a face-to-face SDM session with a clinical pharmacist. Pharmacists used a decision aid from *Healthwise©* to present evidence-based options for diabetes prevention (DPP/lifestyle change, and/or metformin). Pharmacists then provided participants with information on how to enroll in the DPP and/or prescribed metformin, based on participant choice. Participant recruitment, intervention details, and choice outcomes are described in another manuscript.^22, 23^

### Study Sample and Recruitment

For the current study, we recruited from PRIDE intervention participants (*n*=515) who expressed interest in enrolling in the DPP during SDM and who agreed to be contacted for follow-up studies (*n*=359). Participants with missing data (*n* = 33) on race, ethnicity, follow-up weight, etc. or who did not meet inclusion criteria (*n* = 141) were excluded from the sampling frame, leaving 185 eligible participants (Appendix 1). Using purposive sampling, 54 participants were recruited by telephone and 24 interviews were conducted on Zoom due to the COVID-19 pandemic. Telephone recruitment and interviews were done in a sex-concordant manner by the male author/medical student (R. S.) and a female staff recruiter, following a standardized script. Neither had any prior relationship with participants. All interviews were conducted in English, with the exception of one Spanish interview by a native Spanish speaker on the research team. Interviews were recorded and transcribed. Participants received a $30 gift card. The University of California, Los Angeles, Institutional Review Board approved this study (IRB #20-000824).

**Figure 1:**
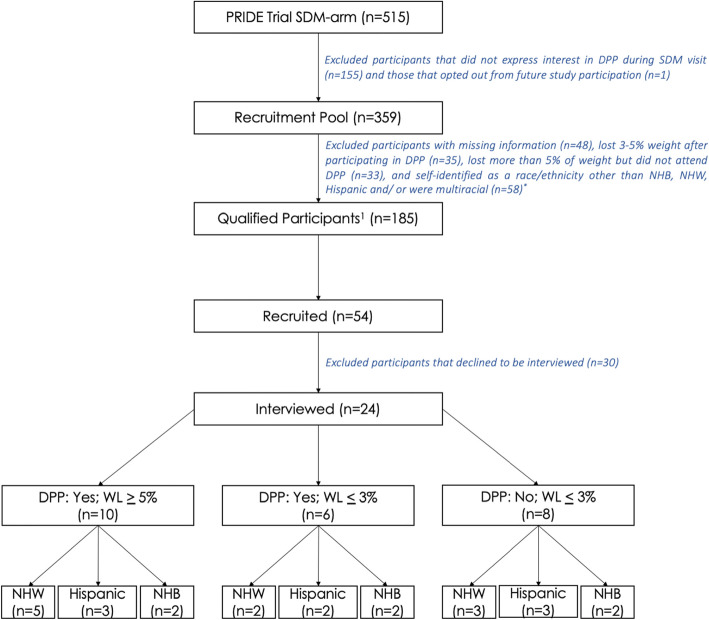
Eligibility and recruitment flowchart. DPP = Diabetes Prevention Program; WL = weight loss; NHW = non-Hispanic White; NHB = non-Hispanic Black. *Multiracial participants who self-reported as non-Hispanic Black/non-Hispanic White and Hispanic/non-Hispanic White were included in the recruitment pool but included in either the NHB or Hispanic category. Participants identifying as Black/Hispanic were excluded from the study.

We classified participants into three groups based on DPP participation and 12-month weight loss: DPP+/WL+ attended >1 DPP session and lost > 5% of body weight; DPP+/WL− attended >1 DPP session and lost < 3% of body weight or gained weight; DPP−/WL− *did not* attend DPP and lost < 3% of body weight or gained weight (Fig. 1). We identified 3% weight loss as a cutoff because studies have shown that patients who exceed this are more likely to maintain weight loss for several years.^24^ We excluded participants who *did not* attend the DPP but lost > 5% of body weight since this group may have lost weight through mechanisms other than lifestyle change (e.g., bariatric surgery). We recruited Hispanic, NHB, and NHW participants from all three groups, yielding nine subgroups based on DPP status, weight loss, and race and ethnicity. Participants identifying as NHW and NHB were classified as NHB, and those identifying as NHW and Hispanic were classified as Hispanic. Participants identifying as both NHB and Hispanic (*n*=5) were excluded from the study as we hoped to examine facilitators and barriers for both groups separately. An average of 2–3 participants were interviewed in each subgroup (Fig. 1).

Semi-structured interview questions were developed based on a social determinants of health framework to elicit barriers and facilitators to lifestyle change and weight loss, and to elicit information about the DPP from program attendees (Appendix 2). Interviews were completed in June/July 2020 and averaged 31 min.

### Data Analysis

Transcripts were coded and analyzed using Dedoose (Dedoose Version 8.0.35, web application for managing, analyzing, and presenting qualitative and mixed method research data [Internet]. Los Angeles (CA): SocioCultural Research Consultants, LLC; c2018). We used grounded theory, which helps develop theories from existing data while conducting comparative analysis, as our guiding framework.^25^ Two researchers independently analyzed and coded each transcript and any discrepancies were discussed in the presence of a third researcher, consistent with a triangulation approach.^26^ Comparisons were made between the groups (e.g., DPP+/WL+ vs. DPP+/WL− vs. DPP−/WL−) and by race and ethnicity, exploring similarities and differences in themes that could contribute to differences in weight loss.

## RESULTS

### Participants

We invited 54 PRIDE participants for interviews and 24 agreed to participate. At the time of the SDM visit, study participants had a mean age of 60 years (range 38 to 73, SD ± 8.7) and 42% self-reported as NHW, 33% as Hispanic, and 25% as NHB (Table 1). Mean weight change at 12 months was −9.3% for DPP+/WL+ (SD ± 4.6), −0.2% for DPP+/WL− (SD ± 2.1), and +1.3% for DPP−/WL− (SD ± 2.9). On average, DPP+/WL+ participants attended 17 DPP sessions while DPP+/WL− participants with available data attended 11 DPP sessions (Table 2).

**Table 1:** Demographic Characteristics of Interview Participants (*n*=24)

ID	Age	Race, Ethnicity and Sex	Educational Attainment
DPP+/WL+ (attended DPP, >5% weight loss)			
B1	52	NHB female	Some college
B2	65	NHB male	College graduate
H1	65	Hispanic male	College graduate
H2	63	Hispanic female	High school graduate or GED
H3	56	Hispanic female	Some college
W1	65	NHW male	More than college degree
W2	61	NHW male	College graduate
W3	71	NHW male	More than college degree
W4	64	NHW male	More than college degree
W5	40	NHW female	College graduate
Mean for DPP+/WL+	**60**		
DPP+/WL− (attended DPP, <3% weight loss)			
B3	62	NHB female	Some college
B4	69	NHB female	Some college
H4	55	Hispanic female	Some college
H5	38	Hispanic male	More than college degree
W6	61	NHW male	Some college
W7	55	NHW female	Some college
Mean for DPP+/WL−	**57**		
DPP−/WL− (No DPP, <3% weight loss)			
B5	65	NHB female	College graduate
B6	47	NHB male	College graduate
H6	57	Hispanic female	Some high school, did not graduate
H7	66	Hispanic male	High school graduate or GED
H8	73	Hispanic male	Some college
W8	58	NHW male	Some college
W9	60	NHW male	More than college degree
W10	62	NHW female	More than college degree
Mean for DPP−/WL−	**61**

**Table 2:** Baseline A1c, DPP Attendance, and Weight Loss of Interview Participants (*n*=24)

ID	Baseline A1c (%)	DPP sessions attended	12-month weight change (%)
DPP+/WL+ (Attended DPP, >5% weight loss)			
B1	6.1	24	−6.8
B2	6.0	15	−6.5
H1	6.1	12	−9.1
H2	6.1	21	−8.5
H3	6.1	25	−5.0
W1	6.4	18	−16.0
W2	6.2	22	−18.9
W3	5.8	21	−5.2
W4	5.8	6	−10.1
W5	5.7	11	−6.8
Mean for DPP+/WL+	**6.0**	**17**	−**9.3%**
DPP+/WL− (attended DPP, <3% weight loss)			
B3	5.8	19	−1.9
B4	5.9	N/A*	−2.6
H4	5.8	12	−0.8
H5	5.8	5	+2.5
W6	5.8	8	−2.1
W7	5.9	12	+1.5
Mean for DPP+/WL−	**5.8**	**11**	−**0.2%**
DPP−/WL− (no DPP, <3% weight loss)			
B5	5.8	0	+2.2
B6	6.4	0	−1.4
H6	6.2	0	+3.4
H7	6.2	0	+5.8
H8	5.9	0	+2.2
W8	6.1	0	−2.0
W9	6.3	0	+4.6
W10	6.1	0	−0.5
Mean for DPP−/WL−	**6.1**	**0**	**+1.3%**

^N^egative values indicate a loss in weight while positive values indicate a gain in weight. *NHW* non-Hispanic White, *NHB* non-Hispanic Black.

*Participant B4 enrolled in DPP and completed the program; however, we could not verify how many sessions she attended. Therefore, she was assigned to DPP+/WL− but was not included in the calculated mean DPP attendance statistics.

### Major Themes

We identified four major themes that facilitated DPP engagement and weight loss: (1) DPP convenience, (2) work/life flexibility, (3) supplemental resources, and (4) social support (Table 3). Among patients who lost 5% body weight, DPP convenience, work/life flexibility, and supplemental resources were more frequently reported by NHW participants as compared to NHB and Hispanic participants, while we did not identify differences by race or ethnicity in Social Support. In this study of patients who are primarily English-speaking with relatively high educational attainment, Hispanic and NHB participants did not report difficulties linked to culture or religion in adopting DPP dietary recommendations.

**Table 3:** Summary of Major Themes Associated with 5% Weight Loss Among PRIDE Trial Participants

DPP convenience	Inconvenient DPP locations and class hours, and limited access to reliable transportation may have impeded weight loss efforts in Hispanic and NHB participants more than NHWs.
Work/life flexibility	Hispanic and NHB participants reported long and inflexible work hours with extended commutes, and had greater difficulties than NHWs in overcoming competing demands and finding time to implement lifestyle change.
Supplemental resources	Participants who successfully lost weight, particularly NHW participants, were able to dedicate additional supplemental resources to support their weight loss efforts.
Social support	Positive and active social support was associated with successful weight loss for all participants, regardless of race or ethnicity.

#### DPP Convenience

Participants who lost weight frequently cited convenient access to the DPP as a contributing factor. This included proximity to the DPP location, being able to manage traffic, and access to reliable transportation, such as “[being] close enough to walk to the sessions” (DPP+/WL+, W2). Some reported challenges that were often surmountable with extra effort. For example, some anticipated difficulty with parking: “I initially was concerned about finding parking … because having gone to [the DPP location], I know the parking situation. But there was available parking just a little walk away” (DPP+/WL+, W1). Others were initially concerned about traffic but noted “[the DPP location] is about 30 minutes away with traffic. But you deal with traffic all the time” (DPP+/WL+, W3).

In contrast, many participants who did not lose weight, especially Hispanic and NHB participants, described transportation barriers that impeded DPP attendance. For example, one described distance as a deterrent to regular DPP attendance as they “didn’t have a car at the time” (DPP+/WL−, B4). Another described, “It was too late for me to go over there on the bus. I think it started at, I don't know, five or six o'clock. And by the time I [would have] left there, it was an hour class or two hours … and then I had to walk a long way to get to the bus, so then when I got off the bus, I had to walk a long way to come to the house” (DPP−/WL−, H8). Another shared a similar sentiment—“I really did want to do that program, but I just felt like it would just be so inconvenient. But at the time, boy, if I had found like a close [DPP] where I live or where my sister lives, I would have done it” (DPP−/WL−, B5). They added, “Where I live, [DPP site] is the closest. But it’s also the most difficult in terms of traffic and parking. If I took the train, then I’d have to hike up a hill, that kind of thing. And I just said that’s not worth it to me” (DPP−/WL−, B5).

Several NHW participants who did not lose weight also cited distance and/or reliance on public transportation as a barrier. One mentioned, “It was a 15-minute drive, and so whatever time I was going to have to take to get there and leave to come back, would just take more time than I wanted to devote to it” (DPP−/WL−, W10). Another said, “The … time to travel from where I live [was a problem] and … I was using public transportation. So that was the inhibitor … the number of bus changes; the amount of time spent on buses back and forth” (DPP−/WL−, W8). Some described a calculation weighing the perceived value of the information versus travel inconveniences. For example, one shared that the “types of [DPP] information that I was [informed] I would learn about was rudimentary or fundamental. It was basic stuff” (DPP−/WL−, W8). When weighed against the challenges of public transportation/distance, DPP enrollment did not seem worthwhile.

#### Employment and Life Flexibility

Work-life flexibility was identified as a common theme facilitating weight loss among DPP participants who lost weight, including being retired or having flexible work arrangements that made it easier to integrate a weight loss routine. For instance, one stated, “Being retired, it’s just like so much easier. It’s just ridiculous [the time commitment of the DPP/weight loss]. Encourage people to quit their jobs or retire” (DPP+/WL+, W4). Among those still employed, one referenced a flexible work structure in helping them maintain their weight loss plan, such as access to exercise equipment on work trips—“I work as a consultant. I was doing traveling … but I still managed to work out … no matter where I go, I always took my bike shorts or shoes so I could ride a stationary bike. I continued working out and watching my diet all the time” (DPP+/WL+, W3). Another participant recalled “At work, my boss let me go to the [DPP] classes. I explained to him what I was doing. And he encouraged me … Actually, at work hours, he let me go for an hour to the class, because … you know, it was concerning to my health… he helped me a lot on that” (DPP+/WL+, H2). In contrast, among participants who did not lose weight, and particularly NHB and Hispanic participants, the lack of work flexibility was perceived as a barrier. One shared that “[they] would have had to take time off work and then try to rush over there. It was just a lot to have to do” (DPP−/WL−, H6).

In addition, participants who did not lose weight, including Hispanic and NHB participants, often reported competing parental demands as a time barrier. One mother recalled, “I have to cook for myself and my kids. I wasn’t able to cook separate because of finances at the time … Were my kids going to be satisfied with what I feed them as opposed to what I could eat to lose weight?” (DPP+/WL−, B4). Similarly, a father stated, “I’ve always been one that has been active … kind of gotten away from it a little bit because of throughout the years of being a single father raising kids, going to work all the time” (DPP−/WL−, B6).

#### Supplemental Resources

Participants who lost weight, particularly NHW participants, described being able to allocate supplemental resources and disposable income to their weight loss effort. Many purchased additional exercise equipment with one saying, “Elliptical machine. I have one at home. I invested in one” (DPP+/WL+, W5). Others talked about investing in “$250 a month gym memberships” (DPP+/WL−, W6) or Pilates classes—“I do $90 private sessions, private one-hour session with a Pilates instructor” (DPP+/WL+, W2). Another participant stated, “I do meals, and it costs me around $75 a week to eat that kind of healthy food all ready. They’re not that expensive. They’re organic and healthy. So, I am still sticking to my diet” (DPP+/WL+, H2). On the other hand, some NHW participants who did not lose weight reported income as a barrier and were not familiar with low-cost lifestyle change options in the DPP curriculum such as walking— “It’s definitely an investment … You have to buy exercise equipment. You have to maybe join a club… You have to invest for your own health, and that will cost you a bit” (DPP-/WL−, W9).

Some participants who did not lose weight, including Hispanic and NHB participants, reported acquiring supplemental resources to support their weight loss efforts. However, compared to the more expensive gym memberships and exercise equipment purchased by participants that lost weight, these participants often reported purchasing less expensive items like athletic shoes or Fitbits. Hispanic and NHB participants frequently cited limited disposable income as a barrier when considering supplemental resources. For instance, one said, “I did pay for a little gym membership and I paid monthly until I couldn’t afford it anymore. So, I wasn’t able to go to the gym and use all of their equipment and their water aerobics and everything” (DPP+/WL−, B4). Hispanic and NHB participants were more likely to rely on insurance-covered services when describing use of supplemental lifestyle change resources such as gym memberships. One describes, “My gym, because I’m 66 years old, and I’ve had this insurance since I was 62. They pay for our gym membership. So, I get the gym membership, so I don’t have to pay for that” (DPP+/WL−, B3). One NHB participant illustrates the challenges faced by low-income participants when seeking supportive lifestyle change resources to encourage weight loss, “I had to learn to do without things. Well, mostly I usually pay my bills, my household bills fully. [Then] I learned to just pay half of it and pay the other half another week. Because I wanted to get the food that helped me get back from [developing] diabetes” (DPP+/WL−, B4).

#### Social Support

Intensive social support from friends and family was commonly reported by participants who lost weight, regardless of race and ethnicity. One commented, “My wife was supportive all the way through. She does most of the cooking, so she just adapted really well to fitting the [recommendations] in. She’s always been kind of health-conscious and everything, so [the DPP] program kind of fed into her interest as well” (DPP+/WL+, W1). Similarily, another added, “With the [DPP] program … I think for me the key thing was my wife learned to shop a new way and prepare food a new way” (DPP+/WL+, H1). In contrast, while participants who did not lose weight sometimes received moderate support such as words of encouragement, others reported receiving negative feedback from their family or friends. A wife noted challenges in cooking for her husband—“he was onboard until he seen his diet was changing too because he is strictly a meat and rice guy, a meat and potato person. But I’m not cooking two different meals. And one night he asks me, ‘Look, I’m not a rabbit. I can’t do all these vegetables. So, can I please have my rice and potatoes or whatever?’” (DPP+/WL−, W7). Some reported feeling stigmatized/judged by their support systems in their weight loss efforts. One commented, “If you don’t eat after church, then they don’t think very well of you. Like, oh, she doesn’t like my food … and then when the lady would go up to get other plates of food to give to us, she would tell her like, ‘Don’t offer [her] anything because she’s not going to eat it and she doesn’t want it. Maybe she doesn’t like it’ (DPP+/WL−, B4).

## DISCUSSION

We identified several themes related to unsuccessful weight loss among Hispanic and NHB participants in our sample, namely DPP inconvenience, limited work/life flexibility, and not purchasing (or inability to purchase) supplementary resources such as exercise equipment. Conversely, many participants who attended DPP and lost at least 5% body weight, particularly NHW participants, commented on DPP convenience, flexibility in their work schedule, and access to supplementary resources as facilitators of success. Social support was also noted as important in successful DPP engagement and weight loss, but this was consistent across racial and ethnic groups.

While PRIDE analyses found that disparities in weight loss persisted after controlling for DPP attendance, more consistent attendance and participation was still associated with improved outcomes.^20^ Other studies have confirmed a positive correlation between DPP attendance, greater physical activity, and more weight loss.^27^ Other work has confirmed racial and ethnic differences in DPP attrition,^15, 19, 28^ with one analysis showing higher DPP retention rates at 18 weeks for NHW participants (71%) compared with NHB (61%) and Hispanic (53%) participants.^28^ Our work suggests that DPP class proximity, access to convenient transportation, and workplace flexibility may partially explain differences in DPP attendance, participation, and weight loss by race and ethnicity. NHB and Hispanic participants may be more likely to have jobs requiring them to be present throughout the workday, making it difficult to participate in the DPP during work hours. They may also depend on public transportation and be reluctant to commit to long bus rides in the evenings after work, particularly if they have responsibilities at home. A recent meta-analysis of 27 weight loss interventions is consistent with our findings, identifying incompatible work structure, lower socioeconomic status, and increased numbers of children at home as correlates of decreased adherence.^29^ DPPs that meet in-person should strive to ensure that their physical location and meeting times are convenient for all participants. Alternatively, recent data supports virtual delivery of DPP as an effective mode to increase DPP engagement.^30^

We found that successful weight loss may be influenced by the availability of disposable income. While PRIDE analyses did not identify differences in weight loss by earned income,^20^ in this follow-up study, we found differences in spending by NHW and minority participants in supporting lifestyle change. Many participants who lost weight, particularly NHW participants, spent discretionary funds for meal preparation services, gym memberships, or physical activity equipment. In comparison, NHB and Hispanic participants were less likely to report similar large purchases and often reported competing financial demands. Of note, some NHW participants reported financial challenges, but they were more likely to fall into the DPP−/WL− group. This is consistent with a recent study highlighting that lower-income NHW participants in the DPP achieve about 25% of the weight loss achieved by higher-income NHW participants.^31^

There is a marked racial wealth gap in the USA due in large part to structural racism and residential segregation, such that individuals from under-resourced populations have more precarious financial situations than NHW individuals with similar incomes. Earned income may be an inadequate measure of the ability to obtain supplemental resources for behavioral change, and less disposable income among NHB and Hispanic participants may explain some disparities in weight loss among DPP participants. Future studies should test this hypothesis by measuring disposable income as a predictor of successful weight loss.

We found that participants from all racial and ethnic groups who lost weight described more meaningful social support. They often had family members who adopted the same behavioral changes, while participants who did not lose weight reported only receiving words of encouragement from family members or friends. Overweight/obese NHB individuals may be more successful in losing weight when they are paired with a family member of similar BMI in a dyadic intervention.^32^ DPPs may be more likely to achieve racial equity in weight loss if they can simultaneously engage individuals and their family members in lifestyle change.

Real-world DPP translations have fallen short in achieving equitable outcomes.^15–20^ While cultural adaptations to the DPP may help to address some racial and ethnic disparities,^33^ future iterations must address the upstream socio-structural determinants that limit minority patients’ access to lifestyle change. For example, US counties with middle-to-high socioeconomic disadvantage have fewer lifestyle change programs and less convenient access to these programs than counties with less socioeconomic disadvantage.^34^ Future solutions to weight loss disparities should attempt to mitigate accessibility challenges through creative modes of DPP delivery. Employing community healthcare workers^35^ and using community and faith-based settings^36^ have proven successful among minority populations. Moreover, new evidence suggests that digital DPP can be an alternative delivery modality for low-income participants.^37^ Low-income participants may benefit from financial incentives to lose weight, including paid time to attend worksite DPP classes.^38^ Finally, health insurance that provides supplemental resources such as free gym memberships may help to bridge gaps and promote health equity.

A recent review outlines a similar argument for supplementing lifestyle change programs such as the DPP with comprehensive strategies that strive for health equity in diabetes prevention. These strategies might include a direct focus on socioeconomic status, living and working conditions, sociocultural context, and sociopolitical context, all of which are important moderators of diabetes prevention.^39^

Our study has limitations, including that qualitative methods are limited when comparing between groups. This project was hypothesis-generating, with limited generalizability to participants outside a large, academic health system. The potential influence of cultural differences in weight loss may not be identified in this highly acculturated population. As our primary interest was disparities among patients who expressed interest in structured lifestyle interventions, we did not interview patients who lost >5% of body weight through other means (e.g., bariatric surgery). Future studies of this population may identify additional salient themes. Finally, recall bias is a potential limitation, since many participants attended/completed the DPP in 2015–2017, up to 5 years prior to this study.

## CONCLUSION

Nearly 88 million Americans have prediabetes, but only 300,000 (~0.3%) have participated in the National DPP to date.^2, 12^ Most (65%) have been NHW while only 21% have been Hispanic or NHB. The need for dissemination and spread of DPP to all populations has received increased attention, which is important. However, it is critical that implementation efforts also focus squarely on achieving racial equity in weight loss outcomes. Increasing the convenience of DPP class offerings, covering supplemental lifestyle change resources through extra health insurance benefits, and encouraging joint participation by close family members and friends are potential strategies to achieve both goals. Systemic adjustments in the national approach to diabetes prevention will be important in decreasing the burden of diabetes while simultaneously promoting equity and justice.

## Supplementary Information

ESM 1 (DOCX 38 kb)
