# Supplementary material for: Differences in Weight Loss by Race and Ethnicity in the PRIDE Trial: a Qualitative Analysis of Participant Perspectives
Source: J Gen Intern Med. 2022 Apr 25;37(14):3715–22. doi: 10.1007/s11606-022-07521-5 (PMC9037581; doi:10.1007/s11606-022-07521-5)
Supplement: Supplementary file 1 — (DOCX 38 kb) [file 11606_2022_7521_MOESM1_ESM.docx]

**Appendix 1:** Racial and Ethnic Distribution of Qualified PRIDE Study Participants (n=185)

|  | DPP+/WL+ (*DPP + / WL > 5%*) | DPP+/WL- (*DPP + / WL < 3%*) | DPP-/WL- (*DPP ­- / WL < 3%*) | Total |
| --- | --- | --- | --- | --- |
| NHB | 6 | 10 | 29 | 45 |
| Hispanic | 9 | 16 | 19 | 44 |
| NHW | 34 | 19 | 43 | 96 |
| Total | 49 | 45 | 91 | 185 |

DPP = Diabetes Prevention Program; WL = Weight Loss; NHW = non-Hispanic White; NHB = non-Hispanic Black.

**Appendix 2:** List of interview questions. DPP+/WL+ and DPP+/WL- participants were asked questions from Sections A and B, while DPP-/WL- participants were asked questions from Sections A and C.

**SEMI-STRUCTURED INTERVIEW**

**A. INTRODUCTORY QUESTION FOR ALL INTERVIEWEES**

1. What reasons originally motivated you to join the study and meet with the pharmacist to learn about your options?
2. When you joined the study, were you concerned about the health consequences of being overweight and at risk for diabetes?  If yes, how concerned were you on a scale of 1 to 5: 1 being no concerned at all and 5 being very concerned.
3. When you joined the study, were you unhappy with the way your body looked, compared to your ideal body size?  If yes, how concerned were you with the way their body looked: 1 being no concerned at all and 5 being very concerned.
4. After meeting with the pharmacist *[Interviewer, specify date of SDM visit here]*, how confident were you in your ability to lose weight? Rank on a scale is 1 being not so confident and 5 being very confident.

*[Interviewer – continue and ask questions in section B if the patient participated in the DPP,* ***or*** *ask questions in section C if the patient did NOT participate in the DPP]*

**B. PARTICIPANTS WHO CHOSE DPP AND PARTICIPATED IN ANY DPP SESSIONS (whether or not they lost >5% body weight)**

1. What motivated you to join the DPP?
2. How much money did the DPP charge you to participate in the program?
3. How important was the cost of the DPP when you considered your decision to join the program? Rank on a scale is 1 being not so important and 5 being very important.
4. How easy/difficult was it for you to find a DPP class that was convenient for you? What types of things made it difficult to find a class (e.g., distance from home or work, day or evening classes, weekend classes, competing demands, etc.)?
5. After attending the first couple of sessions with the DPP, what were your thoughts in being able to adopt the weight loss plans discussed into your life?
6. What were your experiences during the DPP? What aspects did you enjoy?
7. How were you able to adapt the weight loss plans you learned from your DPP coach into your daily life?
8. What was your experience like with the rest of the participants in your DPP class? How well did you get along with the other participants? Did you interact with them during or outside of class?
9. How well did you interact with your DPP coach during/ outside of class time?
10. If you made changes to your eating habits, what kind of changes did you make?
11. If you started or increased your physical activity, what types of activity did you choose? What type of space (e.g. park, home, gym) did you use to be physically active?
12. How effective were you in establishing a daily routine that included your weight loss plan?
13. Were you able to set specific diet and activity goals for yourself, and how well did you stick to those goals?
14. What challenges, if any, did you face in implementing the weight loss plans you learned from your DPP coach into your daily life?
15. Did you find that you had time in your daily life to use the weight loss plans you learned in the DPP?
16. Did you find that you were able to afford the financial cost associated with the weight loss plans you learned in the DPP?
17. Aside from the cost of DPP class, what additional cost did you acquire as a result of your weight loss plan (e.g. travel cost, childcare, physical activity home equipment, modified diet)?
18. How did your family/ friends support you during your time in the DPP? (Ex: financial, words of encouragement, accountability, etc.)
19. [*Interviewer, specify the date of DPP enrollment here].* Were you able to achieve your weight loss goals in the DPP in the first 6 months of the program? How well have you been able to maintain those goals until today?
20. What advice would you give to other people with prediabetes who are trying to lose weight?
21. What racial or ethnic group do you most identify yourself with?  Examples are Black, Hispanic, White, etc.
22. How easy or difficult was it for you to adopt the diet and physical activity strategies from DPP in terms of your own cultural or religious traditions and values?
23. What advice would you have for future programs to adopt the diet and physical activity strategies from DPP, in terms of your own cultural or religious traditions and values?

**C. PARTICIPANTS WHO CHOSE DPP BUT DID NOT PARTICIPATE IN ANY SESSIONS**

1. If you planned to join the DPP when meeting with the pharmacist but later changed your mind, what were the reasons that changed your mind?
2. Were you able to find out how much money the DPP would have charged you to join the program?
3. How important was the cost of the DPP when you considered your decision to join the program? Rank on a scale is 1 being not so important and 5 being very important
4. How easy/difficult was it for you to find a DPP class that was convenient for you? What types of things made it difficult to find a class (e.g., distance from home or work, day or evening classes, weekend classes, competing demands, etc.)?
5. [*Interviewer, specify the date of SDM visit here].* What changes to your diet or physical activity habits did you make after meeting with the pharmacist?
6. How were you able to adapt these weight loss plans into your daily life?
7. If you made changes to your eating habits, what kind of changes did you make?
8. If you started or increased your physical activity, what types of activity did you choose? What type of space (e.g. park, home, gym) did you use to be physically active?
9. How effective were you in establishing a daily routine that included your weight loss plans?
10. Were you able to set specific diet and activity goals for yourself, and how well did you stick to those goals?
11. What challenges, if any, did you face in your pursuit to lose weight?
12. Did you find that you had time in your daily life to use the diet and physical strategies you had planned to adopt?
13. How did your family/ friends support you during your efforts to lose weight? (Ex: financial, words of encouragement, accountability, etc.)
14. Were you able to achieve your weight loss goals in the first 6 months after meeting with the pharmacist?  How well have you been able to maintain those goals until today?
15. What advice would you give to other people with prediabetes who are trying to lose weight?
16. As an estimate, how much did it cost to execute your weight loss plans (e.g. travel cost to parks, purchasing physical activity equipment, dietary modifications)?
17. What racial or ethnic group do you most identify yourself with?  Examples are Black,

Hispanic, White, etc.

1. How easy or difficult was it for you to adopt diet and physical activity strategies in terms of your own cultural or religious traditions and values?
